# Complexation of Anthocyanin‐Rich Blackberry Extract With Ozonated Sodium Alginate: Structural, Rheological, and Color Insights

**DOI:** 10.1111/1750-3841.70869

**Published:** 2026-02-06

**Authors:** Camila Yamashita, Izabel Cristina Freitas Moraes, Charles Windson Isidoro Haminiuk, Ciro César Zanini Branco, Antônio Gilberto Ferreira, Arthur Torres Negreiros, Cassia Roberta Malacrida Mayer, Ivanise Guilherme Branco

**Affiliations:** ^1^ Departamento de Ciências Biológicas Universidade Estadual Paulista (UNESP) Assis São Paulo Brazil; ^2^ Departamento de Engenharia de Alimentos Universidade de São Paulo (USP) Pirassununga São Paulo Brazil; ^3^ Departamento Acadêmico de Química e Biologia Universidade Tecnológica Federal do Paraná (UTFPR) Curitiba Paraná Brazil; ^4^ Departamento de Química Universidade Federal de São Carlos (UFSCAR) São Carlos São Paulo Brazil

**Keywords:** ^1^H NMR, blackberry, depolymerization, encapsulation, FTIR, *Sargassum* spp

## Abstract

Anthocyanins, valued for their health‐promoting properties and their role as natural pigments, are highly susceptible to degradation, highlighting the need for strategies to improve their stability. Biopolymer‐based systems are an alternative for interacting with and safeguarding these bioactive compounds. Ozonated alginate is a potential candidate for complexing with anthocyanins because of its modified structure and properties. This study aimed to evaluate the complexation of anthocyanin‐rich blackberry extract with ozonated sodium alginate (SA) solutions and to compare its properties to those of native (i.e., extracted from seaweed in the lab) and commercial SA. Rheological analyses revealed shear‐thinning behavior in the SA and anthocyanin‐rich extract composite solutions, indicative of their incorporation and molecular entanglement. Structural analyses provided insights into the electrostatic interaction between the biopolymer and the extract, with new peaks and chemical shifts appearing. Microscopy revealed a porous network, suggesting the formation of complexes between SA and the anthocyanin‐rich extract composite solutions. Additionally, the SA and anthocyanin‐rich extract solutions exhibited an attractive color. In conclusion, the complex comprising ozonated SA and anthocyanin‐rich extract has potential applications in diverse sectors, particularly in the food and medical industries, because it leverages the low molecular weight of SA and the bioactive properties of anthocyanins.

## 1 Introduction

Anthocyanins are a subclass of secondary plant metabolites within the flavonoid group, commonly found in a wide range of fruits and vegetables. These natural, water‐soluble pigments display a broad color spectrum—from red to blue depending on pH (Feitosa et al. 2025)—and are associated with various biological and antioxidant activities (Yang et al. 2021). However, their chemical structure is inherently unstable, making them highly susceptible to degradation by environmental factors such as temperature, pH, and oxygen exposure (Liu et al. 2025).

Food polymers can preserve the stability of anthocyanins through the formation of molecular complexes in specific systems (Cortez et al. 2017). Given that anthocyanins are water‐soluble pigments, they are typically anchored to hydrophilic polymers, most notably polysaccharides (Luan et al. 2025). Biopolymer‐based formulations are environmentally friendly, edible, cost‐effective, and easily modified and are ideal for food applications (Luo et al. 2020). Among biopolymers, alginates are linear, anionic, and water‐soluble polysaccharides composed of (1 → 4)‐linked monomeric units of α‐d‐mannuronate (M) and β‐l‐guluronate (G), arranged in homopolymeric M‐blocks, G‐blocks, and alternating MG‐blocks (Kumar et al. 2024). Alginate has shown strong binding affinities to anthocyanins; this parameter is essential for the entrapment of these compounds within the network formed through ionic crosslinking of the polysaccharide (Liudvinaviciute et al. 2020). Moreover, alginate with a higher content of G‐blocks is more favorable for complex formation with anthocyanins, primarily due to electrostatic interactions between its carboxylate groups and the flavylium cations of anthocyanins (Liudvinaviciute et al. 2020; Wang et al. 2025). This interaction contributes to the structural stability of the resulting complexes and plays a crucial role in protecting anthocyanins from environmental degradation.

Alginates are typically extracted in their native form from brown seaweeds such as *Sargassum* species. Their physicochemical properties can subsequently be tailored through chemical or physical modifications to suit specific food and bioproduct applications. Ozone gas, a powerful oxidizing agent, has shown great potential for modifying alginate structure (Yamashita et al. 2021). This technology is environmentally friendly, as it rapidly decomposes into oxygen, leaving no harmful residues in food products or the environment (Ai et al. 2024). Oxidation processes can promote the depolymerization of alginate, resulting in a reduced molecular weight (Mw) and, consequently, lower viscosity (Yamashita et al. 2021), while increasing its chemical reactivity (Reakasame and Boccaccini 2018) with the formation of additional carboxyl groups during oxidation (Ai et al. 2024). Another advantage of this process is its capacity to bleach the native color of alginate while preserving natural antioxidants. This ability is an important benefit for future applications in food‐based products (Yamashita et al. 2021).

Alginate‐based systems have emerged as promising carriers for anthocyanins in functional foods, nutraceuticals, and pharmaceutical applications, where pigment retention is a critical performance parameter (Kumar et al. 2024; Liudvinaviciute et al. 2020). However, few studies have explored the complexation of anthocyanin with modified sodium alginate (SA), despite its promising potential to enhance pigment stability for various industrial applications. Therefore, this study aimed to evaluate the complexation of anthocyanin‐rich blackberry extract powder redispersed in ozonated alginate solutions and to compare its physicochemical, structural, and morphological properties with those of anthocyanin complexes obtained in native and commercial SA solutions.

## 2 Materials and Methods

### 2.1 Extraction of SA From Seaweed

Brown seaweed (*Sargassum* spp.) was collected from Ubatuba, SP, Brazil (October 2022). The algal material was sanitized, dried for 24 h at 45°C in a convection oven (Model 420‐1D, Ethik Technology, Vargem Grande Paulista, Brazil), ground with a cutting mill, and stored in airtight containers at room temperature. The algal powder (7 g) was subjected to two rounds of treatment with 99% ethanol (50 mL for each treatment) with continuous agitation for 3 h, followed by overnight drying in an air circulation oven (Model 420‐1D, Ethik Technology) at 40°C. The pretreated seaweed was subsequently supplemented with a 20% citric acid solution (anhydrous; Synth, Diadema, SP, Brazil) adjusted to a pH of 1.5. The algal suspension was ultrasonicated at a power of 160 W for 9 min using a probe with a 12.7‐mm tip diameter via an ultrasonic horn (20 kHz frequency and 400 W maximum power dissipation; OR‐T‐500, OMNI International, Kennesaw, GA, USA). The samples were rinsed with deionized water and treated with 2% (w/v) sodium carbonate (anhydrous; Impex, Diadema, SP, Brazil) at pH 10 for 90 min at 76°C under magnetic stirring (Nogueira et al. 2022). The mixture was filtered, the supernatant was precipitated with two volumes of 99% ethanol, and the extracted SA was dried at 45°C for 24 h in a convection oven. Hereafter, it is referred to as the native SA (N).

### 2.2 Ozonation of SA Extracted From *Sargassum* spp

The SA aqueous solution (1%, w/v) was placed in a 500‐mL glass container (diameter of 7 cm and height of 20 cm) that was half‐filled by the ozonation reaction. Ozone gas was obtained by the conversion of oxygen to ozone in an ozone generator (Model GOBSUS, OzonioBras, Araçatuba, SP, Brazil). The SA solution was subjected to continuous bubbling of ozone via a bubble diffuser positioned at the inner base of the glass container. The treatment involved exposing the SA solution to a controlled feed gas flow of 1 L/min for 10 min at 25°C. The feed gas flow, consisting of oxygen, was regulated via a pressure regulator, whereas the solution temperature was maintained via a jacket connected to a thermostatic bath. To ensure thorough and consistent exposure of the sample to ozone, the solution was subjected to magnetic stirring. After ozone treatment, the samples were concentrated in a rotary evaporator (60°C), precipitated in two volumes of 99% ethanol, and dried at 45°C for 24 h in a convection oven. After ozonization, the sample was denoted as ozonated SA (O); it presented a viscosity‐average Mw of 39.37 kDa and a mannuronic acid (M) to guluronic acid (G) ratio (M/G) of 0.72. To assess the effects of ozonation, the treated SA was compared with both native SA (Mw of 102.30 kDa and M/G of 0.75) and a commercial low‐viscosity SA (Mw of 46.39 kDa and M/G of 1.10; A1112, Sigma‐Aldrich, St. Louis, MO, USA). Detailed characterization and methods for all types of alginate used in this study are described in Yamashita et al. (2025).

### 2.3 Preparation of Anthocyanin‐Rich Extract Powder

Frozen blackberry (*Rubus* spp.; DeMarchi, Pelotas, RS, Brazil) was stored in a freezing chamber at −10°C and thawed in a refrigerator (7–8°C) for 24 h. Water was added at a 1:3 fruit‐to‐water (w/v) ratio, and the mixture was shaken mechanically at room temperature in the dark for 8 h. The extract was filtered, concentrated using a rotary evaporator at 60°C, until one‐third of the initial volume remained (Souza et al. 2015), freeze‐dried, and stored in dark glass containers.

High‐performance liquid chromatography coupled with diode array detection (HPLC‐DAD/UV–Vis, Prominence, Shimadzu, Kyoto, Japan) was used to identify and quantify the bioactive compounds of the extract. A Hypersil BDS column (C18, 250 × 6 mm^2^) at 35°C was used to separate the compounds. The sample (111 mg) was dissolved in methanol under stirring for 10 min and centrifuged. The supernatant (0.5 mL) was mixed with methanol (0.25 mL) and acetonitrile (0.25 mL) and filtered through a 0.22‐µm pore filter. Subsequently, 10 µL of the sample was injected. The mobile phase comprised solvent A (0.1% phosphoric acid, v/v) and solvent B (acetonitrile). The following gradient was applied: 2% B (0–30 min), 8% B (30–50 min), 30% B (50–52 min), and 2% B (52–62 min), followed by washing and reconditioning of the column. The flow rate was 1.0 mL/min, and the runs were monitored at different wavelengths for phenolic acids and the flavonoids (see Table 1 for the details). Standard calibration curves were used for quantification.

**TABLE 1:** The chemical composition (mg/100 g extract) of freeze‐dried anthocyanin extract from blackberry (*Rubus* spp.).

Group of phenolic compounds	Compound	Retention time (min)	Wavelength (nm)	Concentration (mg/100 g)
**Phenolic acids**	Ferulic acid	46.53	320	34.81 ± 0.1
	Syringic acid	39.42	276	47.27 ± 1.1
**Flavonoids**	Quercetin	59.94	370	7.82 ± 1.3
	Rutin	40.95	246	18.41 ± 8.6
	Isoquercetin	41.75	280	37.88 ± 2.8
	Cyanidin‐3‐*O*‐glucoside	28.17	516	80.27 ± 8.5
	Procyanidin B2	28.17	280	340.88 ± 9.9

### 2.4 Production of SA Solutions Doped With Anthocyanin‐Rich Extract Powder

Aqueous solutions of three distinct forms of SA (ozonated, native, and commercial) were prepared in deionized water at 2% (w/v). The pH was then adjusted to 4.9 ± 0.1 with an HCl (1 M) solution, matching the pH of the commercial SA solution. Anthocyanin powder was added to each SA solution until a final concentration of 100 mg/mL was reached under continuous magnetic stirring overnight.

#### 2.4.1 Colorimetric Analysis

The color of the anthocyanin‐rich extract–SA solutions was quantified using a colorimeter (CR‐5, Konica Minolta, Ramsey, NJ, USA) in accordance with the CIELAB (L*, a*, b*) color space. Key color attributes—including lightness (L*), the chromatic coordinates a* (+*a* = red; −*a* = green) and b* (+*b *= yellow; −*b* = blue), and additional metrics such as chroma (C*) and hue angle (*h*)—were assessed. The samples were diluted at a 1:1 ratio with deionized water because of their low lightness, and all measurements were performed in triplicate. The overall color variation (∆E*) between the commercial samples and the native and ozonated samples was calculated by using the following equation:

(1) $$\begin{array}{ccc} & & \mathrm{Total}\;\mathrm{color}\;\mathrm{difference}\;\left(\Delta E^{*}\right)=\\ & & \sqrt{{\left(L^{*}-{L_{0}}^{*}\right)}^{2}+{\left(a^{*}-{a_{0}}^{*}\right)}^{2}+{\left(b^{*}-{b_{0}}^{*}\right)}^{2}} \end{array}$$

where *L*_0_*, *a*_0_*, and *b*_0_* are the color parameters of the commercial sample, and L*, a*, and b* are the color parameters of the native and ozonated samples.

#### 2.4.2 Rheological Measurements

Steady‐state shear measurements of the SA and anthocyanin extract composite solutions were conducted with a rheometer (TA Instruments, New Castle, DE, USA) using a double concentric cylinder (external radius 17.5 mm, internal radius 16.0 mm, internal radius 15.3 mm, height 56 mm, and gap 2000 µm) at 25°C. Shear stress was determined at shear rates ranging from 0.01 to 300 s^−1^. The Ostwald–de–Waele (Equation 2), Herschel–Bulkley (HB) (Equation 3), and Cross (Equation 4) rheological models were fitted to the experimental data, obtained in triplicate (*n* = 3) for each treatment, and the fitting quality was assessed based on statistical parameters (i.e., the coefficient of determination [*R* ^2^], the residual sum of squares [RSS], and chi‐square [*χ* ^2^]).

(2) $$\tau =K\times \;\gamma^{n}$$

(3) $$\tau =\tau_{0}+K\times \;\gamma^{n}$$

(4) $$\eta =\eta_{\infty }+\frac{\eta_{0}-\;\eta_{\infty }}{1+{\left(\alpha \times \;\gamma \right)}^{m}}$$

In the above equations, τ is the shear stress (Pa), *ϒ* is the shear rate (s^−1^), *K* is the consistency index (Pa s*^n^*), *n* is the flow behavior index (dimensionless), τ_0_ is the yield stress (Pa), *η* is the apparent viscosity (Pa s), *η*_0_ is the zero shear viscosity (Pa s), $\eta_{\infty }$ is viscosity at an infinite shear rate (Pa s), *α* is a time constant with the dimensions of time, and *m* is a dimensionless rate constant.

#### 2.4.3 Fourier Transform Mid‐Infrared‐Attenuated Total Reflectance (FMIR‐ATR) Spectroscopy

The functional groups and bonds of alginates with anthocyanin‐rich extracts were characterized using FMIR spectroscopy (Spectrum One, Perkin Elmer, Waltham, MA, USA), equipped with a Spectrum Universal ATR accessory. The samples were freeze‐dried, and analysis was carried out in the range of 4000–650 cm^−1^ at a scan rate of 32 scans with a 4 cm^−1^ spectral resolution. For comparison purposes, SA without the anthocyanin extract was also analyzed.

#### 2.4.4 Proton Nuclear Magnetic Resonance Spectroscopy (^1^H NMR)

The ^1^H NMR spectra were recorded on an Avance III NMR spectrometer (Bruker, Karlsruhe, Germany) operating at 400 MHz and 80°C. Freeze‐dried samples of pure SA (ozonated, native, and commercial), without and with anthocyanin‐rich extract, were dissolved in deuterium oxide (D_2_O), with the sodium salt of trimethylsilylpropionic acid (TSP‐d4) used as the internal reference. The chemical shifts of the anomeric proton signals, block structure, and M/G ratio were determined using the methodology outlined by Jensen et al. (2015). The data were analyzed with the TopSpin software (version 4.3.0).

#### 2.4.5 Morphological Properties

The morphological properties of the freeze‐dried SA (ozonated, native, and commercial) and anthocyanin‐rich extract composite solutions were analyzed using a scanning electron microscope (EVO MA 15, Zeiss, Oberkochen, Germany). Powdered samples were coated with gold after the tape stubs were attached. The samples were analyzed at an accelerating voltage of 20 kV with 100, 1000, and 5000× magnification.

### 2.5 Statistical Analysis

The data are presented as the mean ± standard deviation from at least three independent experiments. One‐way analysis of variance (ANOVA) followed by the Tukey test was used for comparison among the groups. A *p* value ≤0.05 was considered to indicate a statistically significant. OriginPro 2018 (OriginLab Corporation, Northampton, MA, USA) was used for statistical analysis and to generate graphs.

## 3 Results and Discussion

### 3.1 Anthocyanin‐Rich Blackberry Extract Powder

**FIGURE 1:**
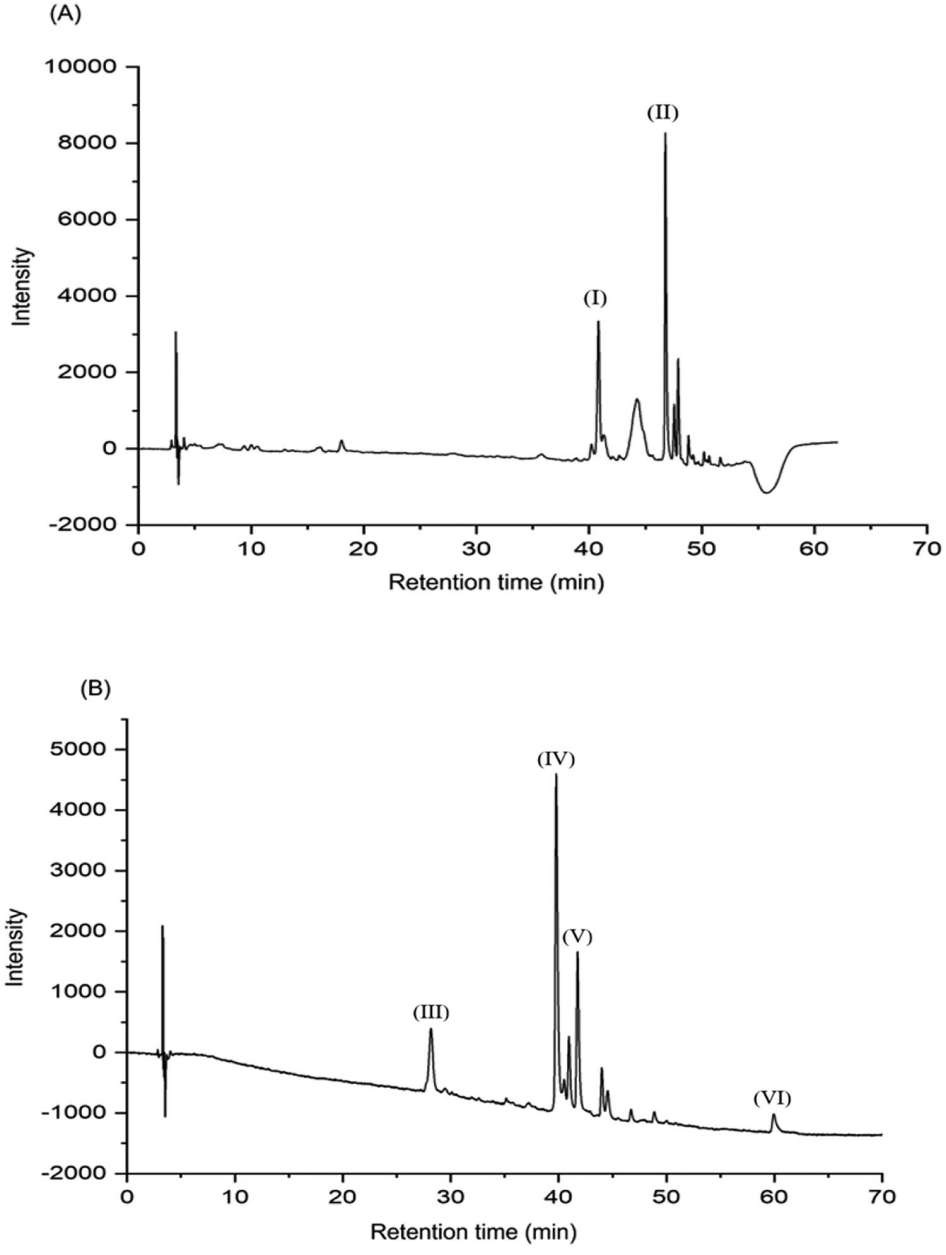
Chromatograms obtained for anthocyanin‐rich blackberry extract showing (A) phenolic acids at 300 nm and (B) flavonoids at 370 nm. The Roman numerals correspond to the retention time of the following compounds: (I) syringic acid, (II) ferulic acid, (III) procyanidin B2 and cyanidin‐3‐*O*‐glucoside, (IV) rutin, (V) isoquercetin, and (VI) quercetin.

Commercial, native, and ozonated SA solutions (2%, w/v) were used to study the formation of complexes with anthocyanins. A commercial blackberry aqueous extract was chosen as the anthocyanin source. The HPLC results (Figure 1 and Table 1) revealed that seven dominant components were present in the anthocyanin‐rich extract, with procyanidin B2 and cyanidin‐3‐*O*‐glucoside being particularly prevalent. These compounds are notably abundant in berries, with anthocyanins comprising aglycones (anthocyanidins) and their glycosides (Padmanabhan et al. 2016). Procyanidin B2 (Table 1) contains four benzene rings and 10 hydroxyl groups and is associated with several biological activities, such as its probiotic effects on the intestinal microbiota prevention of diet‐induced obesity (Xing et al. 2019), promotion of wound healing (Fan et al. 2021), ability to counteract antioxidative stress (Zhuan et al. 2022), and inhibitory effects against cancers (Chen et al. 2023). Cyanidin‐3‐*O*‐glucoside consists of a benzopyran core, a phenolic ring, and a glucopyranoside moiety (Liu et al. 2020). This structural configuration contributes to its physicochemical stability and underlies its diverse biological and therapeutic activities, including potent antioxidant, anti‐inflammatory, and cytoprotective effects (Olivas‐Aguirre et al. 2016). Our results are consistent with the literature, which has reported that the phenolic content in blackberries is 114–1056 mg/100 g of fresh weight (FW), whereas the phenolic acid content in blackberries is 7–64 mg/100 g of FW (Padmanabhan et al. 2016).

### 3.2 Color of SA and Anthocyanin‐Rich Extract Powder Composite Solutions

Color is an important sensory attribute in the analysis of food ingredients. In addition to their previously mentioned health‐promoting attributes, anthocyanins possess an appealing color profile. Consequently, the color indices of the SA complexes were investigated. There was a dominant positive a* value across all formulations. This outcome was predictable because the SA solutions maintained a pH of approximately five before the introduction of anthocyanins; at this pH, there is a predominance of flavylium cations known to favor a reddish hue (Castañeda‐Ovando et al. 2009). The b* values were also positive, indicating yellow coloration. Compared with the native and ozonated SA samples, the commercial SA samples presented higher b* values, indicating a lighter color, which demonstrated a tendency toward a blue hue. This observation was further substantiated by the luminosity (L*): The commercial SA sample displayed the highest value. Importantly, studies have demonstrated that CIELAB coordinates correlate strongly with anthocyanin content in food matrices, suggesting that colorimetric analysis can serve as an indirect predictor of pigment concentration (Vieira et al. 2019). Consequently, the presence of anthocyanins resulted in a more pronounced and vibrant color in the commercial sample, as indicated by the elevated chroma value (Table 2), followed by the ozonated SA composite solutions. The hue angle is used as a color reference, considering the reference 0° or 360° (red), 90° (yellow), 180° (green), and 270° (blue) (Kuck and Noreña 2016). All the samples presented values within the same quadrant, indicating the prevalence of red hues and, thus, the dominance of anthocyanin pigmentation. The hue angle for the commercial sample was the highest, indicative of a less intense color compared with the ozonated and native SA samples, which exhibited similar hue angles. This difference may be explained by the intrinsic properties of each matrix: The commercial SA displayed higher luminosity, which could dilute the perceived intensity of the anthocyanin color, whereas the native and ozonated samples, with their slightly brownish undertones, may have enhanced the red pigmentation through background contrast. These observations align with reports that copigmentation and polymer–pigment interactions can modulate anthocyanin color expression and improve pigment stability (Gençdağ et al. 2022). After each color index was analyzed, it was expected that the ozonated SA sample would exhibit a smaller difference than the native sample, as indicated by the total color difference (∆E*). However, the ozonated SA composite solution presented the second‐best performance after the commercial SA formulation, even exhibiting an inherent brownish color that did not seem to adversely affect the anthocyanin pigment.

**TABLE 2:** The color parameters (CIELAB), chroma (C*), hue angle (*h*), and total color change (∆E*) of the commercial, native, and ozonated sodium alginate with anthocyanin‐rich blackberry extract.

	L*	a*	b*	C*	*h*	∆E*
**Commercial**	10.7^a^ ± 0.05	33.0^a^ ± 0.1	18.0^a^ ± 0.1	37.6^a^ ± 0.2	28.7^a^ ± 0.1	—
**Native**	3.7^b^ ± 0.05	20.6^b^ ± 0.2	6.3^b^ ± 0.1	21.6^b^ ± 0.2	17.0^b^ ± 0.3	18.4
**Ozonated**	4.4^c^ ± 0.04	22.9^c^ ± 0.2	7.4^c^ ± 0.1	24.1^c^ ± 0.2	17.9^c^ ± 0.3	15.9

*Note*: Different letters in the same column indicate significant differences (*p* ≤ 0.05). Data are the mean ± SD (*n* = 3).

### 3.3 Flow Behavior

**FIGURE 2:**
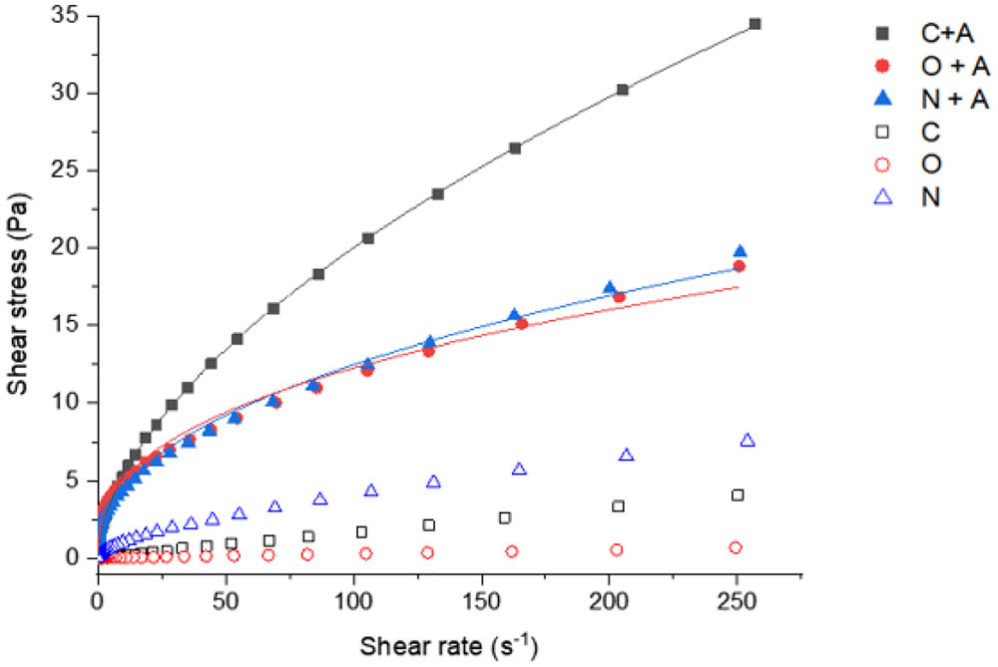
Flow curves of 2% (w/v) pure sodium alginate solutions (C, commercial; O, ozonated; and N, native) and complexed with anthocyanin (A) (C + A, commercial sodium alginate + anthocyanin; O + A, ozonated sodium alginate + anthocyanin; N + A, native sodium alginate + anthocyanin). The continuous lines are fit curves for the power law rheological model.

The flow curves of 2% (w/v) commercial, native, and ozonated SA with and without anthocyanin‐rich extract are presented in Figure 2. Notably, the flow behavior exhibited substantial divergence between the solutions with and without incorporation of the extract. Although the SA solutions displayed Newtonian‐like characteristics (data not shown), those containing anthocyanins showed non‐Newtonian behavior. The shear stress increased with the addition of anthocyanin‐rich extract to the SA solutions. This increase may be due to enhanced intramolecular interactions between SA and the extract (Mogharbel et al. 2024), possibly resulting from the hydrogen bonds formed between polyphenols and SA (Zeng et al. 2021), specifically the –COOH group of SA and the –OH group of extract components (Liu et al. 2012). These interactions likely contribute to a more efficient entrapment of the extract within the polymer network, a phenomenon further amplified by the presence of solid soluble in the samples.

Different models were fitted to the rheological data (Equations (2), (3), (4)). The power law is generally used to fit flow curves for many polysaccharides, such as SA, whereas the HB model is essentially a modified power law with yield stress (Islam et al. 2004). On the other hand, Cross model fluids behave as power law fluids over a range of shear rates and include regions of constant viscosity at a very low or zero shear rate (*η*_0_) and at a very high shear rate (*η_∞_*) (Hauswirth et al. 2020). This model assumes that the pseudoplastic flow is related to the formation and rupture of structural linkages in a material (Afzal et al. 2018). On the basis of the statistical parameters (Table 3), the three rheological models suitably describe the flow behavior of SA with anthocyanin‐rich extracts, as evidenced by the high *R* ^2^ and low RSS and *χ* ^2^ values. As shown in Table 3, all the samples exhibited shear‐thinning fluid behavior (*n *< 1), with the native and ozonated SA doped with the anthocyanin‐rich extract showing greater pseudoplasticity (lower *n* values) than the commercial SA complexes.

**TABLE 3:** Rheological model fitting and statistical parameters of sodium alginate (SA) incorporated with anthocyanin‐rich extract.

Model	Parameters	Commercial + anthocyanin extract	Native + anthocyanin extract	Ozonated + anthocyanin extract
Ostwald‐de‐Waele (power law)	*n*	0.57	0.44	0.38
	*K* (Pa s*^n^*)	1.44	1.66	2.10
	*R* ^2^	0.99	0.99	0.99
	RSS	0.20	6.54	8.05
	*χ* ^2^	0.005	0.15	0.18
Hershel–Bulkley	$\tau_{0}$ (Pa)	0.10	0.68	0.74
	*n*	0.58	0.50	0.45
	*K* (Pa s*^n^*)	1.38	1.15	1.47
	*R* ^2^	0.99	0.99	0.99
	RSS	0.04	1.74	4.02
	*χ* ^2^	0.001	0.04	0.09
Cross	$\eta_{\infty }$ (Pa s)	0.16	0.20	0.20
	$\eta_{0}$ (Pa s)	10,401	92,192	108,782
	(*α* (10^6^ s))	12.3	3.06	5.01
	*m*	0.55	0.72	0.71
	*R* ^2^	0.99	0.99	0.99
	RSS	0.19	3.67	5.23
	*χ* ^2^	0.005	0.087	0.12

The *n* values are lower in the presence of stronger noncovalent interactions due to increased attractive forces between nearby particles, which increase the lifetime of temporary entanglement junctions (Islam et al. 2004). As expected, the consistency index (*K*) was also greater for the native and ozonated SA complexes. The change in apparent viscosity can be attributed to the entanglement–disentanglement process in response to the shear rate, which is related to the number of links and interactions between molecules (Bourbon et al. 2010). Regarding the Cross model parameters, both zero (*η*_0_) and infinite (*η*_∞_) viscosities were elevated for the composite solutions formulated with native and ozonated SA, which can be attributed to the greater interactions between the polymer chains, as discussed above. Overall, *η_∞_* did not differ significantly among the samples, indicating that the internal friction was comparable across all formulations (Nik et al. 2005). The equilibrium time (*α*), marked by the end of zero‐shear viscosity and the onset of shear thinning, was prolonged for the sample formulated with commercial SA, suggesting greater initial structural stability. From a practical perspective, power law behavior during shear flow tests is the most suitable choice. This is primarily because the yield stress (*τ*_0_), which represents the minimum stress required for the material to initiate flow (Barnes 1999), was nearly negligible (<1) (Table 3). Consequently, this model reduces the complexity of the components needed to describe the flow behavior.

This shear thinning behavior is consistent with the observations of Feng et al. (2021), who developed a 3D‐printable ink based on SA and enriched with an anthocyanin‐rich extract from dried rose petals. Their study also revealed an increase in apparent viscosity upon the addition of anthocyanins. Nevertheless, the native and ozonated SA samples exhibited remarkably similar rheological behavior (Figure 2). This suggests that modification of SA derived from seaweed did not yield discernible differences under the evaluated conditions. Despite exhibiting similar rheological properties, when analyzed separately, the commercial and ozonated SA samples demonstrated distinct outcomes when they were complexed with the anthocyanin extract (Figure 2). These variations may be attributed to differences in their chemical structures, thereby influencing their rheological behavior with the formulation of SA and anthocyanin‐rich extract composite solutions.

Structural changes through ozonation did not induce different rheological properties of ozonated SA compared with native SA. However, depolymerized ozonated SA can offer advantages for various applications. For example, Li, Guo, et al. (2023) explored the potential synergistic effects of alginate oligosaccharides and cyanidin‐3‐*O*‐glucoside on gastrointestinal health—a promising application that may be relevant to ozonated SA. Li, Xia, et al. (2023) studied oxidized SA in a formulation with anthocyanins to fabricate films to monitor food freshness. Notably, oxidative treatment via ozone gas has the potential to depolymerize the polysaccharide chains (Yamashita et al. 2021), thus offering promising possibilities for their application.

### 3.4 FMIR‐ATR Spectroscopy

**FIGURE 3:**
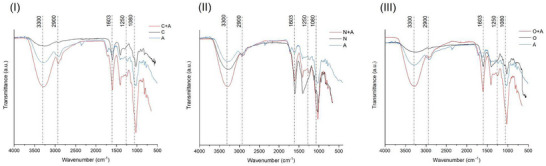
Fourier transform mid‐infrared‐attenuated total reflectance (FMIR‐ATR) spectra of anthocyanin‐rich extracts (A), complexes of (A) and sodium alginate, and their individual forms. (I) C, commercial sodium alginate and C + A, commercial sodium alginate and anthocyanin; (II) N, native sodium alginate and N + A, native sodium alginate and anthocyanin; (III) O, ozonated sodium alginate and O + A, ozonated sodium alginate and anthocyanin.

Infrared spectroscopy provided information about the interactions involved in the complexation of anthocyanin‐rich extract and the SA solutions. The spectra of the separated ingredients and those of SA‐complexed anthocyanin‐rich extract solutions are shown in Figure 3. The spectrum of pure SA showed a vibrational band at 3300 cm^−1^, attributed to the hydroxyl group. However, in the SA solutions containing the anthocyanin extract, the intensity of this band was greater, indicating strong intermolecular hydrogen‐bonding interactions between them (Fan et al. 2022), with a more pronounced difference in samples prepared with ozonated and commercial SA than with native SA. This difference in intensity is most likely due to the presence of aromatic phenols from anthocyanins in SA solutions, where phenolic OH groups enhance the absorption band intensity, as well as intermolecular hydrogen‐bonding interactions (Santiago‐Adame et al. 2015).

There were also differences in intensity, mainly for the commercial and ozonated complexes, in the bands centered at approximately 2900 cm^−1^, which were attributed to the elongation of the C‒H bonds of the pyranoid‐ring carbons, and the 1080 cm^−1^ (C‒O stretching) vibrations of the C‒O‒C glycosidic bonds (Huang et al. 2017; Khajouei et al. 2018). Within the range of 1600–900 cm^−1^, bands associated with phenolic compounds were evident. For example, at approximately 1250 cm^−1^, as depicted in the spectrum of the free extract, the presence of flavonoid tannins, specifically proanthocyanidins, was observed (Ping et al. 2012; Wang et al. 2025). Therefore, the same band appeared in all SA and anthocyanin‐rich composite formulations. Moreover, alterations in the asymmetric and symmetric stretching vibrations of the carboxylate (COO^−^) groups (Borazjani et al. 2017) were discernible, with changes in intensity at 1603 and 1410 cm^−1^ in the SA solutions containing the extract structures, indicating interactions between the compounds. These band changes can be attributed to the electrostatic interactions between the positively charged procyanidin/cyanidin and the negatively charged COO^−^ on SA (Zou et al. 2021).

Alterations in the spectral region around the C═C stretching vibrations of aromatic rings and notable changes in the 750 cm^−1^ region provide additional evidence that anthocyanins were integrated into the SA polymeric matrix; they are indicative of the presence of aromatic rings with *ortho*‐substitution (Pereira et al. 2015). Furthermore, the distinctive peak associated with the stretching vibration of the phenolic hydroxyl group, typically observed at approximately 3600 cm^−1^ for polyphenols, was notably absent in the FMIR‐ATR spectra. This absence suggests the active involvement of the phenolic hydroxyl group of procyanidin in the complexation with SA (Ji et al. 2023). The changes in the intensity and wavenumber of the peaks indicate interactions between SA groups and the anthocyanin flavylium cations (Castañeda‐Ovando et al. 2009; Navikaite et al. 2016) and that the complexation maintained the main structure of SA.

### 3.5 ^1^H NMR

**FIGURE 4:**
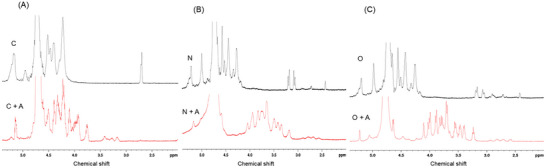
Proton nuclear magnetic resonance spectroscopy (^1^H NMR) spectra of sodium alginate (SA) and anthocyanin‐rich extract (“A”) composite solutions and pure SA dissolved in deuterium oxide (D_2_O) at 80°C. (A) C, commercial SA and C + A, commercial SA and anthocyanin‐rich extract; (B) N, native SA and N + A, native SA and anthocyanin‐rich extract; (C) O, ozonated SA and O + A, ozonated alginate and anthocyanin‐rich extract.

^1^H NMR analysis was conducted to explore the potential interactions between SA and the anthocyanin‐rich extract. In Figure 4, two distinctive peaks are evident, allowing a comparison between the spectra of SA and the combination of SA with the extract. The peak at 4.75 ppm was attributed to the solvent and therefore excluded from the analysis. The spectra of SA without the extract revealed characteristic peaks corresponding to the two uronate residues in SA, assigned at 5.15 ppm (signal of the proton at the 1‐position of the guluronic unit), 4.5 ppm (protons at the 1‐position of the mannuronic unit and the 5‐position of the heteropolymeric sequence of guluronic‐mannuronic units), and 4.4 ppm (proton at the 5‐position of the homopolymeric guluronic block) (Gomez et al. 2007). Remarkably, the SA + extract spectra included cross‐peaks in the chemical shift for the anomeric glucose hydrogens at 4.5 and 5.2 ppm for α‐ and β‐glucose, respectively, whereas other signals of glucose, fructose, and sucrose in the 3.5–4.0 ppm range were incorporated (Pereira et al. 2005). These findings indicate intermolecular interactions between SA and the anthocyanin‐rich extract. Additionally, more peaks in the SA + extract spectra were detected in the range of 3.5‒4.5 ppm, which could be attributed to signals from protons corresponding to the structure of procyanidin (Goncalves et al. 2011), a major component of the extract. Notably, when the anthocyanin‐rich extract was incorporated into both the ozonated and native SA samples, an upfield shift of the protons was observed compared to the spectra of SA without the extract (in pure SA, signals are around 4.5 ppm, and in SA + A, they are around 3.5–4.0 ppm). Another notable signal is a singlet at 3.34 ppm, assignable to methoxyl groups (ferulic acid), which were also present in the anthocyanin‐rich extract (Céspedes et al. 2010).

### 3.6 Morphological Observations

**FIGURE 5:**
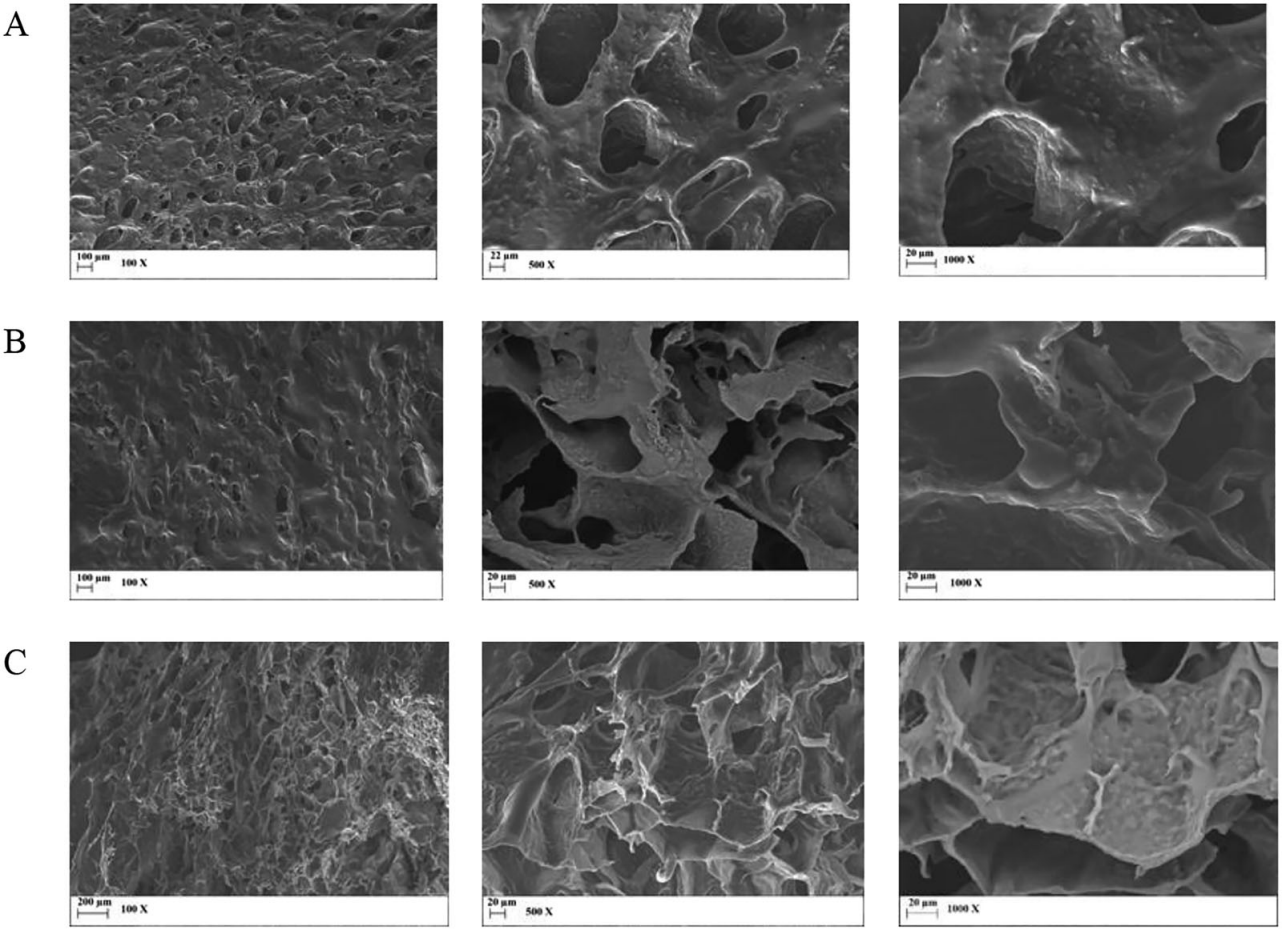
Scanning electron micrographs of (A) native, (B) ozonated, and (C) commercial sodium alginate solutions with anthocyanin‐rich extract.

Scanning electron microscopy was employed to investigate the morphology of anthocyanin‐rich extract complexed in the SA solutions after freeze‐drying. As shown in Figure 5, the particles exhibited porous network structures, indicating that the extract and the SA were connected by fiber‐like bridges that collapsed when the water was removed by freeze‐drying (Bušić et al. 2018; Zou et al. 2021). In addition, the surface of the complex was rough, indicating that the ordered structure of SA was destroyed by the insertion of the anthocyanin‐rich extract through intermolecular interactions (Ji et al. 2023). The morphology and porosity observed in the freeze‐dried samples closely resemble those reported by Zou et al. (2021), which contained cyanidin‐3‐*O*‐glucoside and alginate. Smaller pores were observed in the SA and anthocyanin‐rich extract composite solutions formulated with commercial SA, indicating an improvement in the interaction (Mao et al. 2023), which can enhance the stability of encapsulated bioactive molecules (Meng et al. 2021).

The scanning electron microscopy results revealed that the content of the anthocyanin‐rich extract was adequate; higher concentrations would have led to aggregate (Zheng et al. 2022). All formulations presented irregular, disordered, and dense pores, indicating an intensely unstable and easily destroyed structure (Chen and Zhang 2019). Thus, SA solutions are associated with other components, such as proteins and other polysaccharides, to improve their stability and, consequently, rheological and mechanical properties (Abasalizadeh et al. 2020; Wang et al. 2021).

## 4 Conclusion

This study investigated the formation of complexes between anthocyanins and three types of SA: commercial, native, and ozonated. HPLC analysis identified procyanidin B2 and cyanidin‐3‐*O*‐glucoside as the major anthocyanins in the blackberry extract. The colorimetric properties of the complexes exhibited a dominant positive a* coordinate, indicative of a reddish hue, with the complex formed using commercial SA appearing lighter than the complexes formed with native and ozonated SA. Rheological analysis revealed shear‐thinning behavior in the anthocyanin–alginate systems, which was accurately described by the power law model. The rheological behavior, together with FTIR and ^1^H NMR analyses, indicated strong intermolecular interactions, particularly in the ozonated SA complex, evidenced by changes in vibrational bands and chemical shifts, supporting the occurrence of complexation between anthocyanins and SA. These findings suggest the potential of ozonated SA for preserving and delivering anthocyanin pigments, with promising applications in food and pharmaceutical formulations, particularly where enhanced anthocyanin stability is required.

## Author Contributions

**Camila Yamashita**: conceptualization, data curation, formal analysis, investigation, methodology, validation, visualization, writing – review and editing, writing – original draft. **Izabel Cristina Freitas Moraes**: methodology, formal analysis, validation, writing – review and editing. **Charles Windson Isidoro Haminiuk**: methodology, writing – review and editing. **Ciro César Zanini Branco**: resources, writing – review and editing. **Antônio Gilberto Ferreira**: formal analysis, methodology. **Arthur Torres Negreiros**: formal analysis, writing – review and editing. **Cassia Roberta Malacrida Mayer**: methodology. **Ivanise Guilherme Branco**: conceptualization, data curation, funding acquisition, methodology, supervision, validation, visualization, writing – review and editing.

## Conflicts of Interest

The authors declare no conflicts of interest.
